# A Comprehensive Mapping of the Druggable Cavities within the SARS-CoV-2 Therapeutically Relevant Proteins by Combining Pocket and Docking Searches as Implemented in Pockets 2.0

**DOI:** 10.3390/ijms21145152

**Published:** 2020-07-21

**Authors:** Silvia Gervasoni, Giulio Vistoli, Carmine Talarico, Candida Manelfi, Andrea R. Beccari, Gabriel Studer, Gerardo Tauriello, Andrew Mark Waterhouse, Torsten Schwede, Alessandro Pedretti

**Affiliations:** 1 Dipartimento di Scienze Farmaceutiche, Università degli Studi di Milano, Via Mangiagalli, 25, I-20133 Milano, Italy; silvia.gervasoni@unimi.it (S.G.); giulio.vistoli@unimi.it (G.V.); 2 Dompé Farmaceutici SpA, Via Campo di Pile, I-67100 L’Aquila, Italy; carmine.talarico@dompe.com (C.T.); candida.manelfi@dompe.com (C.M.); andrea.beccari@dompe.com (A.R.B.); 3 Biozentrum, University of Basel, Klingelbergstrasse 50-70, CH-4056 Basel, Switzerland; gabriel.studer@unibas.ch (G.S.); gerardo.tauriello@unibas.ch (G.T.); andrew.waterhouse@unibas.ch (A.M.W.); torsten.schwede@unibas.ch (T.S.); 4 SIB Swiss Institute of Bioinformatics, Biozentrum, University of Basel, Klingelbergstrasse 50-70, CH-4056 Basel, Switzerland

**Keywords:** SARS-CoV-2, pocket search, pocket druggability, docking simulations, virtual screening, blind docking

## Abstract

(1) Background: Virtual screening studies on the therapeutically relevant proteins of the severe acute respiratory syndrome Coronavirus 2 (SARS-CoV-2) require a detailed characterization of their druggable binding sites, and, more generally, a convenient pocket mapping represents a key step for structure-based in silico studies; (2) Methods: Along with a careful literature search on SARS-CoV-2 protein targets, the study presents a novel strategy for pocket mapping based on the combination of pocket (as performed by the well-known FPocket tool) and docking searches (as performed by PLANTS or AutoDock/Vina engines); such an approach is implemented by the Pockets 2.0 plug-in for the VEGA ZZ suite of programs; (3) Results: The literature analysis allowed the identification of 16 promising binding cavities within the SARS-CoV-2 proteins and the here proposed approach was able to recognize them showing performances clearly better than those reached by the sole pocket detection; and (4) Conclusions: Even though the presented strategy should require more extended validations, this proved successful in precisely characterizing a set of SARS-CoV-2 druggable binding pockets including both orthosteric and allosteric sites, which are clearly amenable for virtual screening campaigns and drug repurposing studies. All results generated by the study and the Pockets 2.0 plug-in are available for download.

## 1. Introduction

The exploration of the potential binding cavities within a given target protein represents a key step among the computational tasks, which follow the target identification [1]. After the experimental resolution or the in silico modeling of the target structure, such a mapping enables the identification of the most relevant binding sites for which virtual screening simulations or de novo rational design should allow the identification of promising hits. Clearly, a medium-size protein can have a large number of cavities, which show different shapes (holes, clefts, pores, tunnels, etc.) as well as different roles [2]. Therefore, the first crucial step in the cavity mapping is the recognition of those cavities that are accessible and can have a role in modulating the protein activity. Yet again, not all the potentially interesting cavities can be effectively engaged by interacting ligands. While playing a role in protein activity, some cavities are indeed unable to generate stable complexes with (at least) small drug-like molecules due to their structural, physicochemical, or electronic properties. This fact led to the concept of pocket druggability, the evaluation of which should allow the identification of those cavities for which the hit identification should be reasonably productive [3].

Hence, it comes as no surprise that a remarkable number of computational approaches have been proposed in the last years with a view to mapping the cavities within a given protein structure and to evaluating the resulting druggability. They comprise methods based on Voronoi tessellation [4], grid search [5], surface analysis [6], void sphere clustering [7] or they can involve various combinations of these [8]. Moreover, the increasing number of experimentally resolved protein structures allows a markedly more accurate validation of these methods [9]. By using a purposely collected cavity database, a recent benchmarking analysis compared some well-known approaches for mapping the protein cavities and revealed that FPocket and GaussianFinder are those offering the best performances, with FPocket being able to identify the highest number of correct cavities [10].

Even though the above-discussed methods to detect protein cavities also include an evaluation of their druggability, other methods are specifically focused on the druggability analysis of the found pockets. They can be based on the physicochemical and structural local properties of the cavities or the comparison with homologous proteins or the sole sequence analysis. Often, these methods combine various kinds of information and develop their predictions by using machine learning approaches [11].

To the best of our knowledge and although very often the detected pockets are further evaluated by performing preliminary docking simulations using reference ligands, the combination of pocket searches with systematic docking analyses as an automatic tool to find and to prioritize the target cavities has never been proposed. Therefore, the present study describes Pockets 2.0, a plug-in implemented in the VEGA ZZ suite of programs [12], which combines the FPocket method, chosen due to its notable performances (see above) with docking simulations as performed by PLANTS [13] or by AutoDock/Vina programs [14]. The overall Pockets 2.0 performances were then assessed by comprehensively analyzing a dataset comprising all non-structural or therapeutically promising SARS-CoV-2 proteins for which orthosteric and allosteric binding sites, as well as some potential ligands, were derived by an extensive literature search focused on the homologous proteins. The dataset includes both resolved structures and homology models, thus allowing potential differences in the Pockets 2.0 performances between them to be revealed.

## 2. Results

### 2.1. Overview of the Available Data on SARS-CoV-2 Binding Pockets

Before applying the here proposed method to identify and to assess potentially druggable binding pockets within the SARS-CoV-2 proteins, a careful literature search was performed. When the binding pockets were not directly identifiable by the available structures, they were derived by comparing the considered SARS-CoV-2 proteins with resolved homologous proteins. This study had two primary objectives: 1) finding putative orthosteric and allosteric pockets by which to assess the performances of the proposed method and 2) finding potential ligands (substrates, inhibitors or modulators) to be used as probes during docking simulations. Not to mention that the here collected information is relevant per se, since it affords a better understanding of the catalytic mechanism and (if present) of the allosteric modulation for the analyzed enzymes and can guide the corresponding docking simulations. Hence, this section reports the relevant information acquired during this literature analysis for some relevant SARS-CoV-2 proteins. All the information collected during such a comprehensive literature analysis is compiled in Table 1.

The main protease (3-chymotrypsin like protein, 3CL-PRO) is a key enzyme involved in viral replication and transcription. Hence, this is a very attractive antiviral target and several resolved structures have been already released. Some resolved structures are in complex with inhibitors thus allowing a precise characterization of its binding cavity. Similarly, screening studies proposed some potential 3CL-PRO inhibitors including both covalent and non-covalent ligands [15]. Here, a total of 14 resolved SARS-CoV-2 3CL-PRO structures were analyzed (5R7Y, 5R7Z, 5R80, 5R81, 5R82, 5R83, 5R84, 6LU7, 6M2N, 6M03, 6Y2E [16], 6Y2F, 6Y2G, 6Y84).

The nucleocapsid protein (N-protein) of Coronaviruses is a structural protein, which packs the RNA genome forming a helical nucleocapsid structure or ribonucleoprotein (RNP) complex [17]. Two X-ray structures of SARS-CoV-2 are already available (PDB Id: 6M3M and 6VYO) but in their apo form. However, five N-protein resolved structures from Human coronavirus OC43 (HCoV-OC43) co-crystallized with different ligands within the RNA-binding site (i.e., C5P, 5GP, U5P, AMP, P34 (inhibitor) with PDB Id: 4LMC, 4LM9, 4LM7, 4LI4, 4KXJ, respectively [37]) are available. The binding site is extremely conserved, leading to easy identification of the corresponding pocket in the SARS-CoV-2 protein.

The ADP ribose phosphatase nsp3 of SARS-CoV-2 was crystallized early this year (PDB Id: 6W02) in complex with the substrate ADP ribose, thus allowing a precise characterization of its catalytic cavity. Nsp3 is believed to play key roles in virus replication, which go beyond the simple phosphatase activity, although many of its functions remain to be investigated [38].

Nsp6 is a membrane-spanning protein involved in the compartmentalized viral RNA synthesis [39]. Until now, no crystal structure was resolved but a theoretical model can be generated by de novo modelling. Even though specific information concerning the nsp6 binding sites are not available, a recent analysis of known SARS-CoV-2 mutants highlighted the key role of a cluster composed of aromatic residues and located within the extracellular loop between the first two transmembrane regions in reasonably determining activity and stability of the proteins, since it might be involved in the interaction with the membrane of the endoplasmic reticulum [18]. Moreover, a potent nsp6 inhibitor (K22) was reported for several coronaviruses and its antiviral activity was ascribed to its capacity to interfere with the interaction between nsp6 and the membrane structure [40].

The nsp9 replicase protein of SARS-CoV-2 was recently resolved (PDB Id: 6W4B). This enzyme is believed to bind RNA-single strand in the viral replication complex [19,41]. So far, no proper binding pockets have been identified on this protein. However, the crystal structure of nsp9 SARS-CoV (PDB Id: 1QZ8 [20]) suggests that molecules could bind close to the RNA binding site. In detail, the putative site for inhibitors can be derived by the observation that nsp9 is structurally homologous to the subdomains of serine proteases, in particular to the second domain of coronavirus 3CLpros (PDB Id: 1P9U, in complex with the protease inhibitor PRD_000457) and to the first domain of human rhinovirus 3CLpros (PDB Id: 1CQQ, co-crystallized with RUPINTRIVIR a peptidomimetic inhibitor) [42].

**Figure 1:**
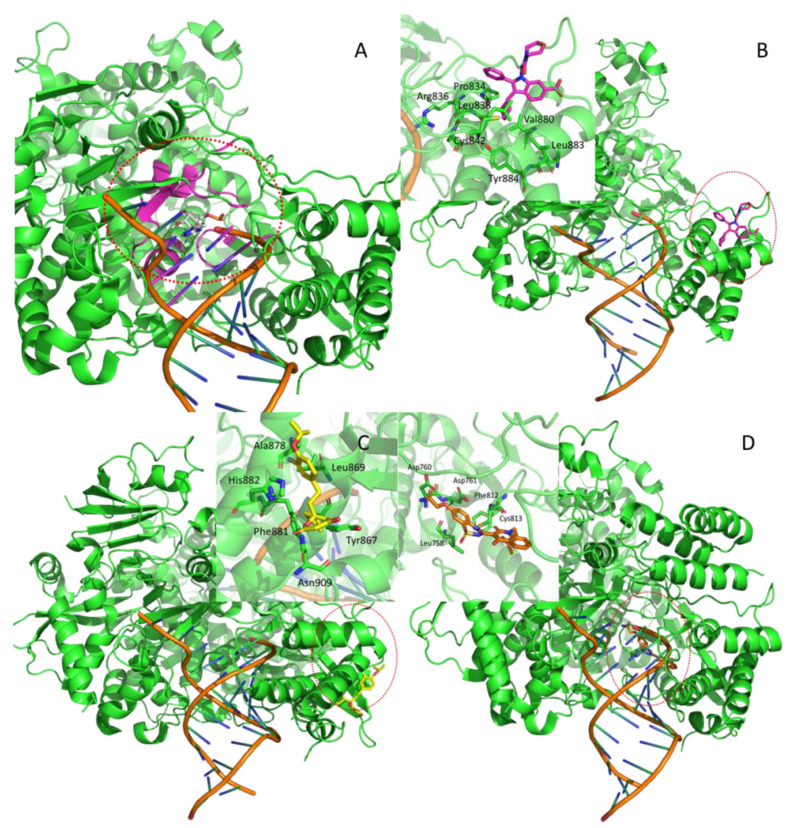
Identified binding sites within the resolved nsp12 structure (PDB Id: 7BV2): the orthosteric cavity in complex with remdesevir (**A**) plus three potential allosteric sites, among which two are located on the “thumb” as defined in their putative complexes with POO (**B**) and PFI (**C**) plus one site on the palm in the predicted complex with 77Z (**D**).

Nsp12 is an RNA-dependent RNA polymerase (RdRp) and is the main enzyme responsible for the RNA replication of the virus, making it an appealing target for the pharmacological treatment of the SARS-CoV-2 infection. Two structures of SARS-CoV-2 nsp12 in complex with its cofactors nsp7 and nsp8 were recently resolved (PDB Id: 6M71 and 7BV2). As depicted by Figure 1, one of these structures (i.e., 7BV2) is co-crystallized with the inhibitor remdesivir, allowing the precise identification of the orthosteric site (Figure 1A). Furthermore, a search for allosteric pockets was also performed since SARS-CoV nsp12 shows structural similarity with the RNA-dependent RNA polymerase of Hepatitis C virus (HCV), called NS5B [43], as both enzymes share the well-known “hand” shape and NS5B is regulated by allosteric modulation [44,45,46]. In detail, three allosteric sites were identified on NS5B: two on the “thumb” (Figure 1B and Figure 1C), and one on the “palm” (Figure 1D). These sites were co-crystallized with three different inhibitors (PDB Id: 2BRL, 2HAI, and 3HHK, Table 1) [21,22,23,47]. All these resolved structures belong to the HCV genotype 1 for which allosteric modulation was best established [48]. Thus, these crystal structures were utilized as a reference for identifying the putative corresponding allosteric sites on the homology model of SARS-CoV-2 nsp12.

The helicase nsp13 catalyzes the unwinding of oligonucleotides duplex into single strands. Given its vital role in virus replication, this has been pointed out as a promising pharmacological target [24]. For this reason, a homology model was built using the crystal structure of MERS-CoV nsp13 (PDB Id: 5wwp [25]) as the template and we looked for possible binding sites. The orthosteric site was retrieved from the resolved structure of the helicase Upf1 from *Saccharomyces Cerevisiae* (PDB Id: 2XZL [26]) which belongs to the Super Family 1 (SF1) of helicases, similarly to SARS-CoV nsp13. Upf1 is in complex with RNA and ADP: AlF4−, which mimics the transition state of the ATP hydrolysis reaction. The orthosteric site is highly conserved and a good agreement between the binding site residues of the crystal structure and the SARS-CoV-2 homology model was found. A putative allosteric site for nsp13 was also derived from the NS3 helicase of HCV (PDB Id: 4B75 [49]). The inhibitor binds at the interface between NS3 and NS4, preventing the interaction between the two proteins. Given the similarity between the structures and the involved residues, a similar allosteric site was also supposed for nsp13, which could be responsible for modulating the interaction with nsp12.

Nsp14 is a (guanine-N7) methyltransferase (N7-MTase) for mRNA capping. Currently, no resolved structure for SARS-CoV-2 nsp14 is available, but the protein can be modeled by homology techniques as the heterodimer nsp10-nsp14, using the available crystal structures of the SARS-CoV nsp10-nsp14 heterodimer (PDB Id: 5C8S [27]) as the template. Luckily, the template was resolved in complex with the functional ligands involved in the catalytic reactions (namely the GpppA substrate and the demethylated SAH cofactor) thus allowing a precise characterization of the catalytic pocket within the modeled SARS-CoV-2 nsp14 structure.

**Figure 2:**
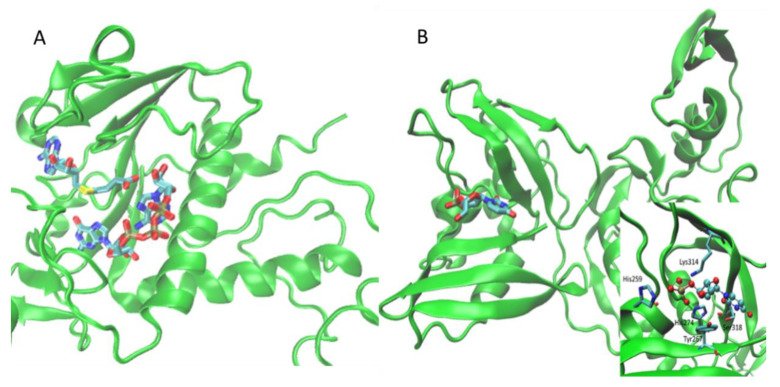
Identified orthosteric cavities within the nsp14 in complex with its ligands, GpppA, and SAH (**A**), as well as within the C-terminal domain of nsp15 in the putative complex with its substrate uridine 3′-phosphate (**B**). In the inlet of 2B, the key contacts stabilizing the complex.

Nsp15 is a uridylate-specific endoribonuclease, the structure of which was recently resolved in complex with citrate (PDB Id: 6W01 [50]). The orthosteric cavity was derived by comparison with the corresponding resolved structures from SARS-CoV (PDB Id: 2H85 [51]). Even though both structures were in their apo form, the orthosteric cavity was identified based on sequence alignment and mutational analyses within the C-terminal domain. As depicted in Figure 2, the so identified cavity was finally verified by preliminary docking simulations with the uridine 3′-phosphate ligand, which produced a pose in encouraging agreement with that proposed for SARS-Cov and Middle East respiratory syndrome coronavirus (MERS-Cov) nsp1. [52,53].

Nsp16 is a 2′ O-methyltransferase [54] and two resolved structures are available in complex (PDB Id: 6W4H and 6WKS) with its cofactor nsp10, which is essential for the activity. The first structure is co-crystallized with SAM, while the second one includes both SAM and the P1-7-methylguanosine-P3-adenosine-5′,5′-triphosphate (GTA) substrate, thus allowing precise identification of the overall catalytic pocket.

The papain-like protease of SARS-CoV-2 (PL-Pro) [28] was recently resolved (PDB Id: 6W9C) in its apo form. A putative binding cavity was derived by the comparison with the corresponding structure from SARS-CoV co-crystallized with a potent inhibitor (PDB Id: 3E9S in complex with TTT).

A last SARS-CoV-2 protein that has attracted great interest in its role in the viral entry is the Spike protein, the receptor-binding domain (RBD) of which is recognized by the human receptor angiotensin-converting enzyme 2 (ACE2) [29]. Hence, different resolved proteins of the sole trimeric SARS-CoV-2 spike protein (PDB Id: 6LVN, 6LXT [30], 6VSB [31], 6VXX [32], 6VYB [33]) as well as of its RBD in complex with ACE2 (PDB Id: 6LZG [34], 6M0J [35], 6M17 [36], 6VW1 [55]) were recently reported. Rather than druggable pockets, these complexes allow a precise characterization of the protein–protein interactions by revealing the key regions involved in spike–ACE2 recognition. In the following docking simulations, the pocket analysis was performed by using mefloquine as the probe ligand since it was recently reported to be able to inhibit the spike–ACE2 interaction [56].

Finally and to the best of our knowledge, no binding sites can be identified by analyzing homologous proteins for the non-structural proteins nsp2, nsp4, nsp7-nsp8, and nsp10. Similarly, the membrane protein (M-protein) and the Open Reading Frame (ORF) proteins (ORF3a, 6, 7a, 7b, 8, 10) are structural proteins for which no druggable site can be found [57].

Such a preliminary analysis allowed the identification of 16 binding pockets within 12 different SARS-CoV-2 proteins: in detail, the orthosteric pocket was identified in all proteins, apart from nsp6 and the Spike protein where the hot spots for the ACE2 interaction were yet detected, an allosteric site was also recognized within nsp6 and nsp13, while at least three allosteric pockets were identified for nsp12. In the following part of the study, the identified binding pockets will be used as a test set to validate the performances of Pockets2.0 by evaluating its capacity to recognize them as the best pocket when ranked by combining pocket and docking searches compared to the two methods taken separately (as reported in Table 2). For simplicity, the identified binding pockets will be named hereinafter as the “correct pockets”.

### 2.2. Pockets 2.0 Performances

As mentioned above, the performances of combining pocket searches and docking calculations were assessed by evaluating the Pockets 2.0 capacity to recognize the 16 previously identified correct pockets as derived from literature search and structural comparisons. Since in several cases, more than one structure was analyzed for each protein (for example, 14 resolved structures were analyzed for 3CL-PRO) and/or more than one ligand was considered for each pocket, this benchmarking study involved 40 pocket searches overall.

Table 2 shows all the investigated proteins with the corresponding detected pockets and the considered probe ligands. Even though the considered 3CL-PRO structures were resolved in complex with different ligands, the pocket searches were repeated by always using the non-covalent inhibitor included in the 6MN2 structure (i.e., 5,6,7-trihydroxy-2-phenyl-4H-chromen-4-one, 3WL) to avoid covalent ligands and to obtain comparable results.

In order to evaluate the correctness of the predicted best pocket, an unbiased approach, which is amenable when analyzing co-crystallized proteins, might be based on rmsd analysis or on volume overlapping between the bound ligand and the pocket shape (as generated by FPocket) or the docked probe molecule (as computed by PLANTS). Unfortunately, the reported analysis involves both theoretical models and resolved proteins with no bound ligands. In these cases, the arrangement of the orthosteric and allosteric binding sites was argued by structural comparison with homologous proteins (as described above) and there are no reference bound ligands for which rmsd or volume overlapping can be evaluated. Hence, the identification of the correct pockets is here based on the identification of some key residues lining the binding site and the detected pockets are evaluated by considering their distance to these key residues. Clearly, the correct pocket is the closest one to the key residues and the binding site is defined as correctly recognized if the closest pocket is ranked as the first pocket (by FPocket, by PLANTS and by consensus score). For example, the performances for 3CL-PRO were evaluated by considering the ranking of the detected pocket closest to the catalytic Cys145 residue.

As explained under Methods, docking simulations were systematically performed within all found pockets using PLANTS. In order to evaluate the Pockets2.0 capacity to recognize the correct pockets, Table 2 also compiles their ranking as based on the FPocket scores, on the docking scores and on the consensus of both scores. For each pocket search, Table 2 reports the volume and the ChemPLP scores as computed by PLANTS for the corresponding correct pockets.

The analysis of the so obtained performances allows for some meaningful considerations. Above all, the combination of FPocket searches and docking calculations leads to a marked improvement in the number of correctly identified pockets since the consensus score recognizes 30 correct pockets out of 40, with an overall satisfactory precision equal to 0.75. Even though the pocket searches involving theoretical models are markedly lower compared to those on the resolved proteins (7 vs. 33), the obtained results do not seem to be influenced by the source of the protein structure since the correct cavity was ranked as best pocket within theoretical models in four cases out of seven.

In detail, FPocket alone was able to recognize one half of the correct pockets (20 out of 40, precision = 0.50), a performance which is in line with that reported by the above mentioned benchmarking study for FPocket when used without repetitions [10]. The performances of docking scores alone are slightly worse than those reached by FPocket (18 out of 40, precision = 0.45) even though it should be noted that docking simulations are not blindly performed on all the protein surfaces but they are focused on the pockets previously detected by FPocket and thus the docking performances unavoidably benefit (at some extent) of the encouraging results offered by FPocket. That being said, one may observe that in 24 cases (out of 40) the rankings of the correct pocket as computed by FPocket and docking simulations do not match and this underlines that docking simulations, while benefitting from the good FPocket performances, encode for additional information that is not simply ascribable to the pocket’s local features.

Interestingly, the consensus score clearly reduces the number of cavities that are incorrectly ranked as second or third cavity compared to the Fpocket or docking scores alone. This indicates that the combination of the two scores allows the pockets that were incorrectly ranked by the two search methods alone to be promoted as the best pocket. In detail, the analysis of the 30 pockets correctly identified by the consensus score reveals that only in 11 cases (out of 11), the correct pockets were ranked as the best cavity by both methods, in 13 cases (out of 16), the correct pockets were ranked as non-best solutions by one method, and in 6 cases (out of 13), the correct pockets were ranked as non-best solutions by both methods. These results reveal that in 19 cases (out of 29), the combination of both methods played a crucial role in identifying the correct pocket, thus emphasizing the remarkable potential of the here proposed combined approach. Intriguingly, the above reported results also allow for another interesting interpretation since they reveal that the correct cavity is successfully recognized in 100% of the cases when both methods ranked it as the best solution. The probability of recognizing the correct cavity decreases to 81% if at least one method ranked it as the best pocket, dropping to 46% if both methods are unable to recognize it as the best solution. These results suggest that the combination of pocket and docking searches affords a mutual validation, which increases the reliability of the predicted pockets.

The validation of the here proposed method can be also assessed by considering that the performed pocket searches involved a total of 1364 cavities, which can be subdivided into 40 correct pockets and 1324 incorrect pockets. This means analyzing confusion matrices including a) 20, 18 and 30 true positives; b) 20, 22 and 10 false positives and false negatives and c) 1304, 1302 and 1314 true negatives for Fpocket, PLANTS and consensus rankings, respectively. While considering such an unbalanced dataset, the increase in the MCC value as obtained by combining pocket and docking search (from 0.48 to 0.74) affords a further confirmation of the potential of the here proposed approach.

A detailed analysis of the investigated proteins reveals that the pocket search for the 14 resolved 3CL-PRO structures is substantially driven by FPocket, which indeed recognizes the correct pocket in 11 cases. These notable FPocket performances can be here explained by considering that its score, while also including parameters related to the local features of the pockets, tends to favor the large pockets. While considering a certain degree of heterogeneity among the reported pocket volumes, which is explainable by considering that even small changes in the arrangement of the lining side chains can induce a different pocket fragmentation by FPocket, the 3CL-PRO catalytic cavity is by far the largest pocket and thus is almost always correctly recognized by FPocket. When discarding the 3CL-PRO structures, the results markedly change since FPocket is able to recognize the correct pocket only in 9 cases out of 26, and the pocket search appears to largely benefit from docking simulations as evidenced by the fact that consensus score correctly recognizes 18 pockets out of 26. These last results emphasize how the combination of pocket and docking searches could be particularly effective when exploring complex proteins endowed with narrow binding cavities, and indeed it appears truly productive when looking for allosteric binding sites, as seen for nsp6 and nsp12.

The analysis of the pockets for nsp12 and the Spike protein deserves a separate description. With regard to the nsp12 allosteric cavities, the two cavities on the thumb region can be identified with difficulty due to the folding of some loops, which partly occupy the cavities, thus leading to their unsatisfactory fragmentation. Hence, the cavity n. 2 was selected since it is large enough to encompass both allosteric sites while not matching either one specifically. Again, the allosteric cavity on the palm region is merged to the orthosteric site, thus generating a unique very large binding site (pocket n. 1), which can suggest the design of bivalent ligands able to interact with both subpockets.

Concerning the Spike protein, all simulations involved the resolved complexes of Spike–ACE2 even though the pockets detected on the ACE2 surface were discarded from the analyses. Though to test the Pockets 2.0 performances, the analysis of the largest Spike–ACE2 complex (PDB Id: 6M17), a hexamer that also includes two chains of the sodium-dependent neutral amino acid transporter B(0)AT1, was also repeated by considering all 275 detected pockets. Gratifyingly, FPocket ranks the correct pocket in the eleventh position while docking simulations proved successful to recognize the correct pocket even in such a challenging situation, and the pocket was ranked as the second one by consensus score, thus further confirming the key role of docking simulations to identify the druggable cavities.

With regard to the badly predicted pockets, there are only two cases in which the correct pocket is ranked off the podium, namely the allosteric site of nsp13 and the orthosteric cavity of the nsp10-14 for the SAH ligand. The unsatisfactory prediction of the nsp13 allosteric pocket can be justified by considering that this binding site should be located at the interface between nsp13 and nsp12 (see above), and thus cannot be properly mapped by considering only nsp13. Again, the wrong prediction for the nsp10-14 heteromer is due to unsuitable docking results, which can be rationalized by considering that SAH is in fact the reaction product and indeed the performances significantly enhance when considering SAM, which is the true cofactor.

## 3. Materials and Methods

### 3.1. Protein Structures

Available experimental structures were downloaded from the PDB (the corresponding PDB Ids are reported in Table 2). The two analyzed homology models for nsp13 and the nsp10-nsp14 heteromer were retrieved by the dedicated SWISS-MODEL page which comprises models for the full SARS-CoV-2 proteome (https://swissmodel.expasy.org/repository/species/2697049). Homology modelling (HM) was performed by SWISS-MODEL [58]. SWISS-MODEL identifies suitable homologues to be used as modelling templates with BLAST [59] and HHblits [60] in the SWISS-MODEL Template Library. Model building using the identified templates and their associated target-template sequence alignments was delegated to the ProMod3 modelling engine (Swiss Institute of Bioinformatics and Biozentrum, Basel, Basel), which performs the following steps: (1) extraction of structural information from the template; (2) loop modelling using an internal loop database or Monte Carlo sampling; (3) side chain modeling using an internal backbone-dependent rotamer library and optimization with the graph-based TreePack algorithm [61] minimizing the SCWRL4 energy function [62]; (4) energy minimization using OpenMM [63] with the CHARMM force field [64]. The global and per-residue model quality of the final models was assessed by the QMEAN scoring function [65] and QMEANDisCo, respectively [66]. The homology models have been deposited in the ModelArchive database with the unique stable accession codes (DOI) https://modelarchive.org/doi/10.5452/ma-epkbe for nsp13 and https://modelarchive.org/doi/10.5452/ma-3rg44 for the nsp10-14 heteromer. De novo models (DN) for nsp6 were obtained from the Feig group (https://github.com/feiglab/sars-cov-2-proteins) [67]. Specifically, the FeigLab and AlphaFold-refined models of nsp6 were used. 

### 3.2. Preliminary Simulations

Be they resolved or modelled, all simulated proteins were prepared by adding (when necessary) the hydrogen atoms to remain compatible at physiological pH and by removing (when present) water solvents, crystallization additives, and bound ligands. The protein structures were then minimized by using Namd2 (NIH Center for Macromolecualr Modeling & Bioinformatics, Urbana-Champaign, Illinois, USA) [68] and by keeping the backbone atoms fixed to preserve the resolved or predicted folding. In detail, the minimizations were carried out by using the conjugate gradient algorithm until RMS = 0.01 kcal mol^-1^Å^-1^ (maximum number of steps = 10000) with the CHARMM force field and the Gasteiger’s atomic charges. All tested probe ligands were optimized by PM7 semi-empirical calculations [69]. When necessary to further assess the pocket reliability (as in the case of nsp15), preliminary docking simulations were performed using PLANTS (Theoretische Chemische Dynamik, Konstanz, Germany) [13] and focusing the search on a 10 Å radius sphere around the identified key residues. For each simulation, 10 poses were generated and ranked by ChemPLP with speed equal to 1.

### 3.3. The Pockets 2.0 Approach

Starting from release 2.4, the VEGA ZZ suite of programs (Drug Design Laboratory, Milan, Italy) comprises a graphical interface to FPocket (named Pocket plug-in) [70]. FPocket (Ressource Parisienne en BioInformatique Structurale, Paris, France) is a well known software to detect protein cavities, which is based on an extremely optimized algorithm for Voronoi tessellation, the performances of which allow even complex macromolecules to be rapidly analyzed [4]. Here a new version of the plug-in for FPocket (named Pockets 2.0) is described. For a better exploration of the protein cavities, this combines the already implemented cavity mapping as performed by Fpocket with docking calculations with probe molecule(s) using AutoDock/Vina or PLANTS docking programs. To optimize the ranking of the explored cavities, Pockets 2.0 can utilize both Fpocket and docking scores by calculating customizable consensus scores.

**Figure 3:**
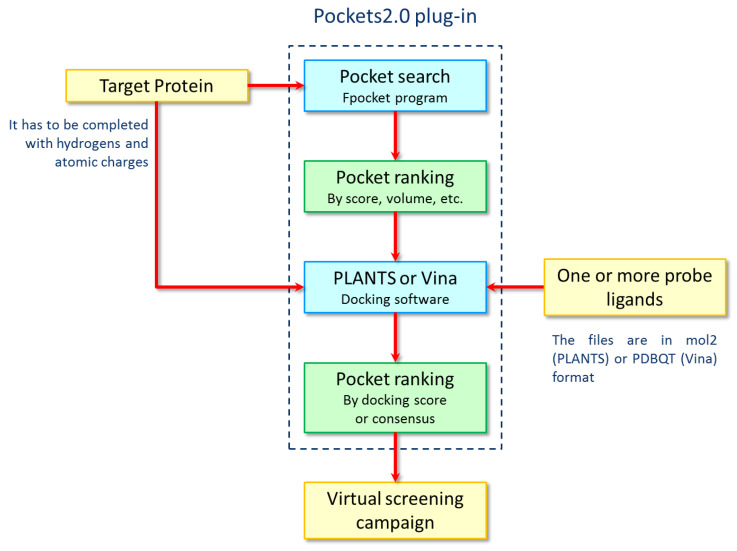
Flowchart describing the main logical steps implemented by Pockets 2.0 to identify and to evaluate druggable protein cavities.

Figure 3 shows the Pockets 2.0 workflow for the identification and prioritization of the potential binding sites for a given protein. The plug-in accepts as primary input the 3D coordinates of the target protein, which can be in any file format supported by the VEGA ZZ program. The input protein structure is firstly analyzed by FPocket, which finds and ranks the cavities according to its internal score. Along with the scores computed by FPocket, the plug-in adds information concerning the geometric features of each pocket, which will be useful for the following docking calculations.

Once the pockets are calculated and analyzed, they can undergo the docking simulations. To do this, the selected ligand(s) should be suitably prepared depending on the chosen docking program. After setting the docking parameters, the simulations are automatically performed on all selected pockets using all collected probe ligands. Based on all computed scores, user-defined consensus functions can be calculated to afford a combined ranking of all cavities. In the here described analysis of the SARS-CoV-2 proteins, the consensus score is computed by summing the ranks obtained by sorting the overall FPocket scores and the ChemPLP docking scores from best to worst.

Along with the interactive inspection of the found pockets and the corresponding ligand poses within the VEGA ZZ graphical interface, the Pockets 2.0 plug-in generates a set of additional output files regarding the computed poses for each pocket and the corresponding score values.

### 3.4. Pocket Search and Analyses

For the here performed analyses, the pocket search was performed by applying the default settings of FPocket (e.g., minimum and maximum radius of alpha spheres equal to 3 Å and 6 Å, respectively) with the only exception of the minimum number of spheres to define a pocket that was set equal to 30 (instead of 35) for a more exhaustive analysis even of the small cavities. For each protein, all found cavities underwent docking simulations using PLANTS. In detail, the search was focused within the corresponding spheres as derived from FPocket analysis by increasing their radius of 3 Å to completely encompass the entire binding cavity. For each simulated ligand and each explored cavity, one pose was generated by using the PLANTS_ChemPLP scoring function with speed equal to 1. In all the analyses, the consensus function of each pocket was computed by combining the overall FPocket score with the ChemPLP score. When more than one ligand was used as a probe for a pocket, the consensus score involves the average of the corresponding ChemPLP scores. Since almost all the analyzed SARS-CoV-2 proteins are enzymes with various catalytic activities, the performances of the here presented combined approach was preliminarily assessed, also considering a set of resolved GPCRs, the evaluation of which was reported in the Supporting Information. As summarized in Table S1, the obtained results also afford an encouraging validation with this class of proteins since the combination of pocket and docking search was able to identify the correct pocket with an overall precision equal to 0.91, even greater than that obtained with the SARS-CoV-2 targets.

## 4. Conclusions

The study had two primary objectives: characterizing the druggable binding sites within the therapeutically relevant SARS-CoV-2 proteins as well as presenting and validating a novel strategy to search and prioritize the protein pockets by using the identified SARS-CoV-2 sites as a test set. Concerning the first objective, an extensive literature search combined with the comparison with homologous proteins allowed precise identification of 16 druggable pockets within 12 different SARS-CoV-2 proteins, including four allosteric sites. In this context, nsp12 represents a very interesting target since its homology with the corresponding HCV enzymes allowed the identification of at least three allosteric pockets, which can play key roles in determining the nsp12 activity as well as its interactions with the cofactor proteins.

Concerning the second objective, the study reports the Pockets 2.0 plug-in as implemented in the VEGA ZZ suite of programs, which automatically combines the pocket search, as performed by FPocket, with docking simulations by using representative probe ligands and PLANTS or AutoDock/Vina as docking engines. The combination of the FPocket and docking scores by calculating customizable consensus scores leads to a significant increase in the correctly identified binding sites compared to the FPocket and docking scores alone, and this enhancement appears to be truly relevant when considering the MCC values which, while considering the dataset unbalancing, show an increase of 35% when combining pocket and docking searches.

Again, an ancillary analysis involving an extended set of aminergic resolved GPCRs (see Supporting Information) revealed that the here proposed approach provides even better performances with this class of therapeutically relevant proteins. Clearly, these encouraging results should be corroborated by more extended analysis, including different proteins of increasing complexity, to verify the general applicability of this strategy. Notably and since docking simulations can involve more than one ligand, Pockets 2.0 can be also used as a powerful tool to perform focused blind docking simulations by which even extended ligand datasets can be simultaneously docked into different potentially relevant binding pockets.

All prepared protein and ligand structures, as well as all files generated by Pockets 2.0 searches, are available at http://www.exscalate4cov.network. The repository will be periodically updated by including the pocket mapping for the new available target proteins. The homology models have been deposited in the ModelArchive database with the unique stable accession codes (DOI) https://modelarchive.org/doi/10.5452/ma-epkbe for nsp13 and https://modelarchive.org/doi/10.5452/ma-3rg44 for the nsp10-14 heteromer. The Pockets 2.0 plug-in is freely available within the VEGA ZZ suite of programs at http://www.vegazz.net.

## Figures and Tables

**Table 1:** Orthosteric and allosteric binding sites for the therapeutically relevant protein targets as derived from the literature search.

Protein	Source ^a^	Function	Reference Protein	PDB Id	Site	Ligand ^c^	Ref.
**3CL-PRO**	See Table 2	protease	---^b^	---^b^	orthosteric	3WL	[15,16]
**N-Protein**	6M3M 6VYO	Nucleocapsid protein	HCoV-OC43 N-NTD	4LMC	orthosteric	C5P	[17]
				4LM9		5GP	
				4LM7		U5P	
				4LI4		AMP	
				4KXJ		P34	
**Nsp3**	6W02	ADP ribose phosphatase	---	---	orthosteric	ADP	*** ^d^
**Nsp6**	DN	Membrane-spanning protein	No experimental information apart from mutants analysis	---	allosteric	K22	[18]
**Nsp9**	6W4B	Replicase	Coronavirus NSP9	1QZ8	orthosteric	SO4	[19]
			Type 2 rhinovirus 3C protease	1CQQ	orthosteric	AG7	[20]
**Nsp12**	7BV2	RNA-dependent RNA polymerase (RdRp)	---	---	orthosteric	F86	***
			Hepatitis C RdRp	2BRL	allosteric1 (thumb)	POO	[21]
			Hepatitis C NS5B polymerase	2HAI	alllosteric2 (thumb)	PFI	[22]
			Hepatitis C NS5 polymerase	3HHK	allosteric3 (palm)	77Z	[23]
**Nsp13**	HM	Helicase	RNA-Dependent ATPase Upf1	2XZL	orthosteric	ADP-ALF	[24]
			Hepatitis C virus NS3 protein	4B75	Allosteric	4VA	[25]
**Nsp14**	HM	Methyltransferase	SARS-CoV	5C8S	orthosteric	SAH, G3A	[26]
**Nsp15**	6W01	Endoribonuclease	SARS-CoV	2H85	orthosteric	U3M	[27]
**Nsp16**	6WKS 6W4H	Methyltransferase	---	---	orthosteric	SAM, GTA	***
**PL-pro**	6W9C	Papain-like protease	SARS-CoV	3E9S	orthosteric	TTT	***
**Spike**	Xray with ACE2	Viral entry glycoprotein	---	---	Protein–protein interaction	YMZ	[28,29,30,31,32,33,34,35,36]

^a^ For resolved proteins, the corresponding PDB Id is reported, while for modelled structures, HM and DN mean homology modeling and de novo modeling, respectively. ^b^ --- means that the binding site was directly identified from the resolved protein SARS-CoV-2 structure. ^c^ The IUPAC name of the probe ligands can be found in the Abbreviations. ^d^ *** indicates that the resolved SARS-CoV-2 protein structures are available in PDB, while the corresponding paper is still unpublished.

**Table 2:** Results of the pocket analysis as performed by Pockets 2.0 on the therapeutically relevant protein targets. The first part of the Table includes data concerning the overall search (such as the source of the analyzed protein, the probe ligand and the number of found pockets), while the second part comprises data concerning the search for the correct pocket. In detail, the Table reports how the correct pocket was ranked by FPocket and PLANTS alone and by consensus score, which combines both searches. The volume and the ChemPLP score for the correct pocket are also listed.

Protein	Protein Data			Data for the Search of the Correct Pocket				
	Source/ID	Ligand	N Pockets	Rank by Fpocket	Rank by PLANTS	Rank by Consensus	Volume (Å^3^)	ChemPLP (kcal/mol)
**3CL-PRO**	5R7Y	3WL	16	1	1	1	3604.93	−76.15
	5R7z	3WL	16	1	1	1	4246.06	−73.85
	5R80	3WL	20	2	2	1	1833.23	−76.48
	5R81	3WL	17	1	1	1	3141.06	−80.27
	5R82	3WL	19	1	4	1	3989.84	−68.23
	5R83	3WL	21	1	1	1	4079.17	−77.59
	5R84	3WL	21	1	1	1	2562.02	−80.88
	6LU7	3WL	14	1	1	1	4239.62	−77.83
	6MN2 dimer	3WL	80	1	1	1	3344.72	−86.30
	6M03	3WL	24	3	3	3	2175.81	−69.70
	6Y2E	3WL	18	1	4	1	2629.29	−75.02
	6Y2F	3WL	20	1	1	1	2581.33	−77.61
	6Y2G	3WL	16	2	3	2	5074.55	−75.78
	6Y84	3WL	20	1	2	1	3068.30	−73.94
**Nsp3**	6W02	APR	6	2	1	1	2247.84	−119.05
**Nsp6**	DN	K22	25	3	1	1	2364.82	−75.26
	DN		27	4	1	1	3692.30	−76.86
**Nsp9**	6W4B	AG7	13	2	3	1	2740.53	−97.41
**Nsp12-Nsp7-Nsp8**	7BV2 trimer	ATP (ortho)	79	1	2	1	4397.28	−94.18
		POO/ PFI (allo)		2	1	1	3545.24	−97.80
		77Z (allo)		1	4	2	4397.28	−85.22
**Nsp13**	HM	ADP (ortho)	40	3	2	2	4359.36	−79.98
		4VA (allo)		5	10	5	2526.96	−74.28
**Nsp14-Nsp10**	HM dimer	SAH	49	2	9	4	5307.98	−80.25
		SAM		2	2	1	5307.98	−88.09
		G3A		2	1	1	5307.98	−119.09
**Nsp15**	6W01 hexamer	U3P	170	5	3	1	2792.58	−80.39
	6VWW dimer	U3P	52	6	1	3	2275.76	−81.75
**Nsp16-Nsp10**	6WKS dimer	SAM	63	1	8	1	4772.88	−88.43
		GTA		1	1	1	4772.88	−118.80
	6W4H dimer	SAM	31	1	1	1	3243.06	−87.81
		GTA		1	2	1	3243.06	−112.33
**N-protein**	6M3M	C5P, 5GP, U5P, AMP, P34	7	3	2	2	1252.56	−66.69
	6VYO		7	3	3	3	1961.99	−70.57
**PL-Pro**	6W95	TTT	23	3	2	1	2134.23	−94.17
**SPIKE–ACE2**	6LZG dimer	YMZ	56	2	2	1	4792.66	−80.66
	6M0J dimer		58	1	5	1	4407.92	−74.29
	6M17 hexamer		275	1 11	1 1	1 2	3767.99	−81.79
	6VW1 dimer		61	1	2	1	4920.74	−77.78
**Correctly Identified Pockets**				20	18	30		
**Correct Pockets Ranked as #2**				9	10	5		
**Correct Pockets Ranked as #3**				6	5	3		
**Correct Rockets Out of the Podium**				5	7	2		
**Average Rank**				2.18	2.43	1.45		
**Precision**				0.5	0.45	0.75		
**Accuracy**				0.97	0.97	0.99		
**MCC**				0.48	0.43	0.74		

## Supplementary Materials

The following are available online at https://www.mdpi.com/1422-0067/21/14/5152/s1, **Table S1:** Results of the pocket analysis as performed by Pockets 2.0 on the collected GPCR targets. The Pockets 2.0 performances were evaluated by considering its capacity to identify the orthosteric and allosteric sites within the selected GPCR complexes.

## Abbreviations

3WL	5,6,7-trihydroxy-2-phenyl-4H-chromen-4-one
4VA	(2S)-4-amino-N-[(1R)-1-(4-chloro-2-fluoro-3-phenoxyphenyl)propyl]-4-oxobutan-2-aminium
5GP	guanosine-5’-monophosphate
77Z	2-({(3R)-3-[(3S)-1-(3-methylbutyl)-2,4-dioxo-1,2,3,4-tetrahydroquinolin-3-yl]-1,1-dioxido-3,4-dihydro-2H-1,2,4-benzothiadiazin-7-yl}oxy)acetamide
ACE2	angiotensin-converting enzyme 2
AG7	4-{2-(4-fluoro-benzyl)-6-methyl-5-[(5-methyl-isoxazole-3-carbonyl)-amino]-4-oxo-heptanoylamino}-5-(2-oxo-pyrrolidin-3-yl)-pentanoic acid ethyl ester
C5P	cytidine-5’-monophosphate
G3A	guanosine-P3-adenosine-5’,5’-triphosphate
GTA	P1-7-methylguanosine-P3-adenosine-5’,5’-triphosphate
HCV	Hepatitis C virus
K22	(Z)-N-(3-(4-(4-bromophenyl)-4-hydroxypiperidin-1-yl)-3-oxo-1-phenylprop-1-en-2-yl) benzamide
MERS-Cov	Middle-East Respiratory Syndrome Coronavirus
MGT	7N-methyl-8-hydroguanosine-5’-triphosphate
N7-MTase	N-7 Methyl transferase
NSP	Non-structural protein
ORF	Opening Reading frame
P34	2-(dimethylamino)-N-(6-oxo-5H-phenanthridin-2-yl)acetamide (PJ34)
PFI	(6S)-6-cyclopentyl-6-[2-(3-fluoro-4-isopropoxyphenyl)ethyl]-4-hydroxy-5,6-dihydro-2h-pyran-2-one
PL-Pro	Papain-like protease
POO	3-cyclohexyl-1-(2-{methyl[(1-methylpiperidin-3-yl)methyl]amino}-2-oxoethyl)-2-phenyl 1H-indole-6-carboxylic acid
RBD	receptor binding domain
RdRp	RNA-dependent RNA polymerase
SAH	S-Adenosyl homocysteine
SAM	S-Adenosyl methionine
SARS-CoV	Severe acute respiratory syndrome coronavirus
SARS-CoV-2	Severe acute respiratory syndrome coronavirus 2
TTT	5-amino- 2-methyl-N-[(1R)-1-naphthalen-1-ylethyl]benzamide
U3P	uridine-3’-monophosphate
U5P	uridine-5’-monophosphate
