# Supplementary material for: A Comprehensive Mapping of the Druggable Cavities within the SARS-CoV-2 Therapeutically Relevant Proteins by Combining Pocket and Docking Searches as Implemented in Pockets 2.0
Source: Int J Mol Sci. 2020 Jul 21;21(14):5152. doi: 10.3390/ijms21145152 (PMC7403965; doi:10.3390/ijms21145152)
Supplement: Supplementary file 1 [file ijms-21-05152-s001.pdf]

Supporting Information

A systematic mapping of the druggable cavities within the SARS-CoV-2 therapeutically relevant proteins by combining pocket and docking searches as implemented in Pockets 2.0

Silvia Gervasoni [1], Giulio Vistoli [1], Carmine Talarico [2], Candida Manelfi [2], Andrea R. Beccari [2], Gabriel Studer [3,4], Gerardo Tauriello [3,4], Andrew Mark Waterhouse [3,4], Torsten Schwede [3,4] and Alessandro Pedretti[1,*]

[1] Dipartimento di Scienze Farmaceutiche, Università degli Studi di Milano, Via Mangiagalli, 25, I-20133 Milano, Italy; silvia.gervasoni@unimi.it (S.G.); giulio.vistoli@unimi.it (G.V.);

[2] Dompé Farmaceutici SpA, Via Campo di Pile, 67100, L'Aquila, Italy; carmine.talarico@dompe.com (C.T.); candida.manelfi@dompe.com (C.M.); andrea.beccari@dompe.com (A.R.B.);

[3,4] Biozentrum, University of Basel, Basel 4056, Switzerland and SIB Swiss Institute of Bioinformatics, Basel 4056, Switzerland; gerardo.tauriello@unibas.ch (G.T.); gabriel.studer@unibas.ch (G.S.); andrew.waterhouse@unibas.ch (A.M.W.); torsten.schwede@unibas.ch (T.S.);

* Correspondence: alessandro.pedretti@unimi.it; Tel.: +39 02 503 19332 (A.P.)

Ancillary analysis on resolved GPCR targets

Since almost all the SARS-CoV-2 proteins analyzed in this study are enzymes, the performances of the Pockets 2.0 tool was preliminarily assessed by considering a second set of proteins composed by resolved GPCRs. Even though an exhaustive validation of the presented approach would require a truly extended set of heterogeneous protein targets, this second ancillary analysis has the objective to test the approach with a completely different class of therapeutically relevant targets with a view to verifying if Pockets2.0 further confirms the encouraging performances obtained by the SARS-CoV-2 proteins.

In detail, this second analysis involved a set of resolved GPCR complexes extracted from the GPCRdb database (https://www.gpcrdb.org/) by applying the following criteria: a) belonging to aminergic class A receptors; b) presence of a co-crystallized ligand; c) resolved the by X-ray technique; d) resolution lower than 3.0 Å. In this way, a set of 43 complexes was collected and underwent the same computational protocol already described for the SARS-CoV-2 proteins.

Table S1 compiles the obtained results for this class of protein targets and reveals overall performances even better than those reached by the explored SARS-CoV-2 proteins. In more detail, Table S1 shows that the combination of pocket and docking search allows the identification of the correctly identified pocket in 39 cases out 43 with an overall very satisfactory precision equal to 0.91. Notably, the docking simulations here perform better the pocket mapping as evidenced by the number of correct pockets (36 vs 33) as well as that of the markedly wrong results (out of the podium) (0 vs. 3) and this gratifying result can be ascribed to the use of resolved complexes the binding sites of which are suitably arranged to accommodate the probe ligands. Taken together, the results for this second analysis afford a truly encouraging confirmation of the potential of the here proposed method as well as of the synergistic role of combining pocket and docking searches for a more accurate characterization of the druggable binding sites of a given protein. Finally, the overall encouraging performances reported by this study should be clearly reinforced by analyzing more extended databases of protein cavities. However, the satisfactory results afforded by two completely different classes of protein targets represent a valuable starting point which is indicative of a general applicability of the reported Pockets 2.0 tool.

Table S1: Results of the pocket analysis as performed by Pockets 2.0 on the collected GPCR targets. The Pockets 2.0 performances were evaluated by considering its capacity to identify the orthosteric and allosteric sites within the selected GPCR complexes.

PDB ID	Ligand	Resolution (Ã)	Fpocket	PLANTS	Consensus
2VT4	P32	2.70	1	1	1
2Y00	Y00	2.50	1	1	1
2Y01	Y00	2.60	1	2	1
2Y02	WHJ	2.60	7	1	4
2Y03	SFW	2.85	1	1	1
3D4S	TIM	2.80	3	2	2
3NY8	JRZ	2.84	1	1	1
3NY9	JSZ	2.84	1	2	1
3PBL	ETQ	2.89	1	1	1
3ZPQ	XF5	2.80	1	1	1
3ZPR	3WC	2.70	1	1	1
4AMJ	CVD	2.30	1	1	1
4BVN	P32	2.10	1	1	1
4IAQ	2GM	2.80	1	1	1
4IAR	ERM	2.70	3	1	1
4IB4	ERM	2.70	2	1	1
4LDE	P0G	2.79	1	1	1
4NC3	ERM	2.80	4	1	2
4U15	0HK	2.80	1	1	1
5A8E	XTK	2.40	1	2	1
5CXV	0HK	2.70	1	2	1
5D5A	CAU	2.48	1	1	1
5DSG	0HK	2.60	1	1	1
5TVN	7LD	2.90	2	2	1
5WIU	AQD	1.96	1	1	1
5WIV	AQD	2.14	2	1	1
5X7D	8VS	2.70	1	1	1
5X7D	CAU	2.70	4	1	2
5YC8	3C0	2.50	1	1	1
5ZK3	QNB	2.60	1	1	1
5ZKB	82F	2.95	1	1	1
5ZKC	3C0	2.30	2	1	1
6A94	ZOT	2.90	1	1	1
6BQH	E2J	2.70	1	1	1
6CM4	8NU	2.87	1	1	1
6DRY	H8D	2.92	1	2	1
6H7J	5FW	2.80	1	1	1
6H7L	Y00	2.70	2	1	1
6H7M	68H	2.76	1	1	1
6H7N	FVK	2.50	1	1	1
6H7O	P32	2.80	1	1	1

6IBL	H98	2.70	1	1	1
6MXT	KSY	2.96	1	1	1
**Correctly identified pockets:**			33	36	39
**Correct pockets ranked as #2:**			5	7	3
**Correct pockets ranked as #3:**			2	0	0
**Correct pockets out of the podium:**			3	0	1
**Average rank:**			1.488372	1.162791	1.13953488
